# Y It Matters—Sex Differences in Fetal Lung Development

**DOI:** 10.3390/biom12030437

**Published:** 2022-03-11

**Authors:** Mandy Laube, Ulrich H. Thome

**Affiliations:** Department of Pediatrics, Division of Neonatology, University of Leipzig, 04103 Leipzig, Germany; ulrich.thome@medizin.uni-leipzig.de

**Keywords:** sex differences, fetal lung development, preterm infants, respiratory distress, epithelial Na^+^ transport

## Abstract

Within this review, sex-specific differences in alveolar epithelial functions are discussed with special focus on preterm infants and the respiratory disorders associated with premature birth. First, a short overview about fetal lung development, the challenges the lung faces during perinatal lung transition to air breathing and respiratory distress in preterm infants is given. Next, clinical observations concerning sex-specific differences in pulmonary morbidity of human preterm infants are noted. The second part discusses potential sex-specific causes of pulmonary complications, including pulmonary steroid receptors and local lung steroid metabolism. With regard to pulmonary steroid metabolism, it is important to highlight which steroidogenic enzymes are expressed at which stage during fetal lung development. Thereafter, we review the knowledge concerning sex-specific aspects of lung growth and maturation. Special focus is given to alveolar epithelial Na^+^ transport as a driver of perinatal lung transition and the sex differences that were noted in this process.

## 1. Background

### 1.1. Fetal Lung Development

Fetal lung development is a highly coordinated process of branching morphogenesis, angiogenesis, and alveolarization that continues postnatally [1]. Therein, two separate consecutive phases can be identified, lung growth representing the structural development (branching morphogenesis) and lung maturation which refers to the functional development. Branching morphogenesis is influenced by several physical factors, including fetal breathing movements, pulmonary fluid secretion, and thoracic volume. In contrast, lung maturation with its functional development is primarily a biochemical process guided by hormones such as glucocorticoids, sex steroids, prolactin, insulin, catecholamines, and several growth factors including fibroblast-pneumocyte factor (FPF) and epidermal growth factor (EGF) [2]. Importantly, branching morphogenesis antagonizes alveolar maturation as shown in the mouse lung [3]. Thus, both processes cannot proceed concurrently.

In addition to the phases of growth and maturation, lung development is further divided into five stages. During the embryonic phase, the lung buds originate as an outgrowth from the ventral wall of the embryonic foregut where lobular division occurs. Subsequently, conducting epithelial tubes are formed by progressive airway branching in the pseudoglandular stage starting at about 5 weeks of human gestation. The canalicular stage takes place between the 16th and 25th weeks during which the gas exchange portion of the lung is formed and vascularized. The saccular stage covers the period from 26 weeks until near term. During this stage, most peripheral airways form widened airspaces, termed sacculi, coated with alveolar type I (ATI) and type II (ATII) cells. The primary septa between the sacculi are still bulky and contain two networks of capillaries. At the end of this stage, the interstitial fibroblasts start to produce extracellular matrix, collagen and elastin, and all generations of the conducting and respiratory branches have been generated. During the last few weeks of pregnancy new sacculi, and from them the first alveoli, form, which are delimited by secondary septa. This alveolarization and therein the formation of secondary septa constitutes the alveolar stage which continues postnatally.

### 1.2. Perinatal Lung Transition

**Figure 1:**
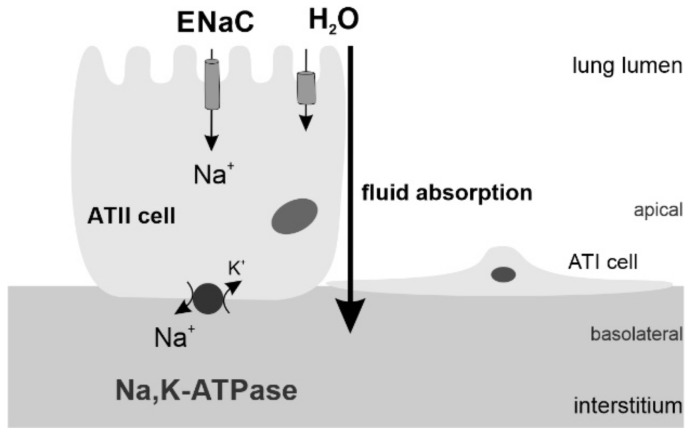
Alveolar epithelial Na^+^ transport. Na^+^ ion are passively taken up by the ENaC in the apical membrane of alveolar epithelial cells and actively extruded by the Na,K-ATPase in the basolateral membrane compartment. Fluid follows this vectorial Na^+^ transport and is absorbed into the circulation.

Intrauterine lung growth is promoted by active fluid secretion [4]. To enable air breathing, the lung has to switch from fluid secretion to fluid absorption before birth. This alveolar fluid clearance (AFC) is accomplished by mature alveolar epithelial cells through unidirectional Na^+^ transport. Apically expressed epithelial Na^+^ channels (ENaC) and the basolateral Na,K-ATPase create a driving force for fluid absorption (Figure 1). In addition, airway pressure induced by screaming, grunting or by mechanical devices contributes to AFC. Mature ATII cells produce and secrete surfactant into the lung lumen where it reduces surface tension within terminal airways and alveoli [5]. Surfactant is a mixture of phospholipids, neutral lipids, and proteins that form a layer between the terminal airways/alveolar surfaces and the alveolar gas [5]. Insufficient expression of Na^+^ channels [6] and surfactant deficiency, as well as structural lung immaturity, impairs AFC and alveolar stability which impedes postnatal lung function and gas exchange.

### 1.3. Respiratory Distress in Preterm Infants

Infants born before the 36th week of gestation are defined as preterm infants, affecting approximately every 10th newborn. Thus, preterm infants constitute the largest pediatric patient group. The limit of viability representing the earliest gestational age at which the infant has at least a 50% chance of survival has been reduced to approximately 24 weeks [7]. Preterm birth is frequently associated with respiratory failure due to structural and functional lung immaturity. The essential events of late-stage lung development include the formation of alveoli and secondary septation, as well as increased production of surfactant. Premature birth disrupts late-stage lung development and thus respiratory distress syndrome (RDS) is frequently observed in preterm infants. RDS is the most common cause of respiratory failure in preterm infants and is inversely related to the gestational age at birth, resulting in a risk of >60% for infants born at 29 weeks, 20% at 34 weeks, and 5% at 37 weeks or term [1]. The pioneering research efforts of Mary Ellen Avery enabled the discovery of surfactant deficiency as the main cause of RDS in preterm infants [8]. Surfactant deficiency causes atelectasis, which in combination with small respiratory units leads to unventilated alveoli resulting in hypoxia, hypercapnia, and acidosis [1]. However, the risk of RDS in infants born at 34 weeks with apparently mature pulmonary surfactant production can reach 8–20% [1,9], indicating that other factors, such as insufficient AFC, play a role in RDS development [10]. Indeed, nasal epithelial potential difference was lower in infants with RDS than in those without RDS [11]. Furthermore, inhibition of the potential difference by amiloride, an antagonist of ENaC, was lower within the first 24 h of life, in infants in whom RDS developed than in those without RDS [11].

### 1.4. Sex Differences in Pulmonary Disorders of Prematurity

**Figure 2:**
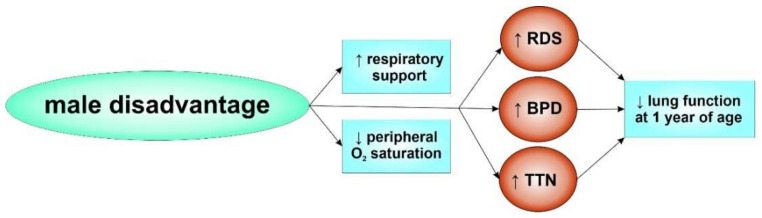
Male disadvantage. The male sex represents an important risk factor for pulmonary complications associated with preterm birth. ↑ represents an increased need or risk, while ↓ describes a reduction.

It has been more than 40 years since sex differences in the incidence of RDS were first noted [12]. Importantly, male preterm neonates exhibit RDS almost twice as often compared with female preterm neonates of the same gestational age (1.7:1) [13]. This sex imbalance, also termed the “male disadvantage”, has still not been resolved and male sex remains an independent risk factor for RDS development, regardless of gestational age [14,15,16,17,18] (Figure 2). In fact, the “male disadvantage” in respiratory illness is not restricted to very early gestational ages or extremely low birth weight but has also been observed in late preterm and term infants [14,15]. Consequently, male mortality was shown to exceed female mortality during the first week of life in infants born between 24 and 32 weeks of gestation, which was mainly related to RDS [19,20]. Supplemental oxygen by mechanical ventilation is frequently required to treat RDS-associated hypoxia and preterm boys need more respiratory support than girls [19,21]. These sex differences persisted in former preterm infants as shown by a follow-up study of lung function at 1 year of age [22].

Apart from RDS, other respiratory diseases are also more common in boys than in girls. Several studies suggest that premature male infants are more prone to the development of bronchopulmonary dysplasia (BPD), a chronic lung disease of prematurity [16,21,23,24]. In this regard, male sex has been shown to be an independent predictor for the development of BPD [25,26]. In a large European cohort of preterm infants with a gestational age below 32 weeks, male sex was a major risk factor for BPD development compared with females [27], confirming previous reports from the Vermont Oxford Network [28]. Furthermore, transient tachypnea of the newborn (TTN) occurs in about 1 out of 100 term neonates. The rate of TTN substantially increases with the growing number of caesarean sections, often performed without preceding labor, raising the relevance of TTN during the past decade [29]. Delayed resorption of fetal lung fluid constitutes the major pathophysiological factor of TTN [30]. Several studies identified male sex as a significant risk factor for TTN development [15,29,31,32]. Female infants were shown to achieve higher peripheral oxygen saturation earlier than males, further confirming a “male disadvantage”. At 4 min after birth, more than 50% of female infants had a peripheral oxygen saturation ≥90%, while only 3% of male infants achieved this saturation [33]. In accordance, lower flows in boys were observed in lung function studies in full-term infants [34,35,36]. Moreover, flows were up to 30% lower in preterm males compared with females, a greater effect than in full-term infants [35,37]. Thus, the “male disadvantage” in respiratory distress is heightened by prematurity [36]. In a twin pregnancy, the respiratory and neurological outcome was enhanced when the fetus (male or female) shared the womb with a female co-twin [38].

Surfactant replacement improved mortality rates of prematurity, but sex differences in survival persist [39]. The viability of extremely low birthweight infants in the postsurfactant replacement era again demonstrated a deleterious effect of male sex on morbidity and mortality [39,40]. Furthermore, male infants require more doses of surfactant than female infants [19,21]. Antenatal administration of glucocorticoids (GCs) to mothers at risk for premature birth reduces RDS incidence by enhancing fetal lung maturation. Whether male and female infants equally benefit from this treatment has been discussed controversially. Some studies suggest that antenatal GCs reduce RDS incidence more in female preterm infants [41,42,43,44], while other studies suggest equal benefits in males and females [16,45]. The incidence of RDS after maternal treatment with betamethasone was 40.9% in males and 7.1% in females [46]. One clinical survey demonstrated that prevention of RDS with betamethasone was far more effective in female infants, worsening the male to female incidence of RDS from 1.7:1 to 3.4:1 [47]. Furthermore, a sex-dependent differential benefit between betamethasone and dexamethasone has been discussed [45].

## 2. Causes

Sex differences in fetal lung development have been observed as early as 16–20 weeks of gestation, showing that fetal mouth movements occur more frequently in girls [48]. Furthermore, the female lung is smaller and has fewer respiratory bronchioles at birth [49], while this is not the case for alveolar numbers per unit. The lung of premature female rabbit fetuses has been shown to be more stable than in premature males [50]. Female preterm lambs were ventilated at lower ventilatory pressures with equivalent tidal volumes, suggesting a more efficient gas exchange [51]. In agreement, a higher percentage of male preterm lambs was dependent on supplemental oxygen and showed a higher inspiratory effort and lower lung compliance than age-matched females [52]. Neonatal male and female mouse pups exposed to hyperoxia demonstrated a sex-specific modulation of angiogenesis and inflammatory responses in the lung [53]. After hyperoxia, male juvenile mice displayed higher lung injury, neutrophil infiltration and apoptosis, indicating that male mice are more prone to hyperoxic lung injuries than female mice [54]. Moreover, a greater improvement for respiratory mechanics was achieved in female preterm lambs in response to steroid treatment [51].

The fetus is exposed to high levels of estrogen (E) and progesterone (P) during late gestation, which decreases by several orders of magnitude after parturition [16]. Premature birth deprives the newborn of female sex hormones at an earlier stage of lung development. Plasma E levels were lower in preterm infants with RDS compared with infants who did not exhibit RDS [55]. In 1979, it was first reported that maternal administration of estradiol (E2) increased concentrations of surfactant phospholipids in fetal rabbit lung lavage [56]. In agreement, direct E injection to the amniotic sac has been shown to stimulate lamellar body formation and release in the fetal rat lung [57]. Furthermore, postnatal E2 treatment in a primate model of BPD improved oxygenation and ventilation indices [58]. Several clinical studies analyzed the replacement of E/P in extremely premature infants who were at risk for BPD [58,59,60]. E/P replacement in 83 extremely premature infants (<29 weeks and <1000 g birth weight) showed a trend toward prevention of BPD or death [61,62], however administration of E/P in neonates attempted for treatment of BPD showed no benefit [59].

Nevertheless, sex steroids are likely involved in the “male disadvantage”. Notably, fetal serum E and P levels are similar in males and females as they are determined by the maternal placenta, whereas fetal rat testosterone (T) levels were higher in males than in females [63]. T is the main circulating androgen secreted by testicular Leydig cells in males, but T is also secreted by ovaries and in small amounts by the adrenal gland [64]. Furthermore, aromatization of T leads to formation of E as wells as dihydrotestosterone (DHT) [65,66]. Thus, T levels per se might not be decisive.

### 2.1. Pulmonary Steroid Receptors

Expression of all sex steroid receptors has been observed in male and female lung tissue [65,66]. Effects of E are exerted by genomic pathways via its receptors ER-α and/or ER-β, which translocate into the nucleus upon ligand binding and subsequently bind to specific DNA response elements to modulate gene expression [67]. Both ERs are present in the lung, although ER-β is more abundant than ER-α [68]. ERs can act as homo- or heterodimers, and ER-α can also heterodimerize with the androgen receptor (AR) resulting in a modified transcriptional activity [69]. The two ERs possess equal binding affinities towards E, but when both ERs are co-expressed in a cell, ER-β can act as a dominant repressor of ER-α function [70]. Notably, the abundance of ER-β in different tissues varies during development [71]. In the fetal lung several studies showed expression of ER-β, while ER-α expression was rarely detected [72,73]. Furthermore, sex differences in ER-β mRNA expression were observed in primary rat fetal distal lung epithelial (FDLE) cells of the saccular stage with males exhibiting lower levels compared with age-matched females [72]. In addition, ER-β immunoreactivity was detected by embryonic day (ED)15 in female lung tissues of fetal mice, one day earlier than its expression in fetal male mice [74]. In addition to the classical genomic pathway, another type of genomic activity occurs through interactions with transcription factors such as c-fos/c-jun [71]. Nongenomic ER signaling involves interactions with cytoplasmatic signal transduction proteins such as mitogen-activated protein kinases (MAPK), extracellular signal-regulated kinases (ERK)1/2, p38 MAPK, phosphoinositide 3-kinase (PI3K), c-Jun N-terminal kinases (JNK), signal transducer and activator of transcription (STAT), and members of the proto-oncogene tyrosine–protein kinase Src family [71,75,76]. Activation of these signaling pathways can subsequently modulate steroid receptor activation, such as ligand-independent activation or direct phosphorylation of steroid receptors [16,77].

Two progesterone receptor (PR) isoforms have been described: PR-B and the N-terminally truncated PR-A, which are both derived from a single gene. Both isoforms have similar ligand binding affinities, but exert distinct functions [78]. PR-B is a strong transcriptional promotor, whereas PR-A is a functional repressor on PR-B and other steroid receptors [78,79,80]. Furthermore, PR-B can crosstalk with ER and thereby activate the MAPK pathway [81]. PRs are further able to induce and regulate multiple cellular signaling pathways independent of nuclear activation [82], such as p42 MAPK and PI3K as shown in Xenopus oocytes [83]. In the fetal lung, PR-A expression was demonstrated, while PR-B expression was not observed [84,85]. Steroid receptor levels can vary considerably within hours, e.g., PRs can be upregulated by E2 and downregulated by P, and ERs are negatively regulated by their ligands [86]. No sex differences were observed in the levels of P or in the abundance of PRs in the fetal rabbit lung at the beginning of the saccular stage [87]. In contrast, male FDLE cells of the saccular stage exhibited lower PR-A levels compared with age-matched females.

Two isoforms of the classic AR have been described (AR-A and AR-B) [88]. The precise role of AR-A remains to be determined, although AR-A has been proposed to antagonize AR-B [89]. ARs also belong to the classical nuclear receptor family, but rapid, non-genomic effects of androgens via membrane receptors have also been suggested [90]. Ligand binding to AR can either activate or inhibit androgen-responsive elements of target genes [91]. T showed a trend towards reducing renal AR mRNA expression in female rats, while AR expression was increased in male rats [92]. Sex hormone-binding globulin receptor possibly mediates the AR-independent effects of androgens and has been observed in the fetal lung [93]. Furthermore, AR is a kinase substrate and a downstream target of receptor tyrosine kinases, e.g., HER-2/neu and G-protein coupled receptor (GPCR) signaling, which can both activate ARs independent of androgen binding [69]. To this end, AR activity is induced by phosphorylation though kinases like MAPK, ERK, p38, JNK, PI3K or protein kinase B (AKT) [69]. AR nongenomic action originates at the plasma membrane or in the cytoplasm, inducing release of intracellular calcium and activation of MAPK pathways [94,95]. The AR is located in the X chromosome and is expressed at similar levels in lung homogenates of both sexes [96] No sex differences were observed in the binding affinities or concentrations of AR in the late gestation fetal rabbit lung [87]. Furthermore, a negative relationship between gestational age and AR expression was reported in fetal humans [97], and AR expression was higher in the early fetal lung than in the adult lung [98]. More precisely, AR expression was demonstrated in fetal mesenchymal cells [99], while another study showed AR immunoreactivity predominantly in epithelial cells of budding sites during fetal lung branching morphogenesis [100,101]. In contrast, no AR-specific mRNA expression was detected in male or female FDLE cells of the saccular stage [102].

Finally, two receptor isoforms have been observed for the glucocorticoid receptor (GR). In human pulmonary epithelial cells GR-α represents the predominant isoform, exerting steroid-binding activity. In contrast, GR-β displays no ligand-binding activity [103] and inhibits the transcriptional activity of GR-α [104]. Mice lacking intracellular GRs die of respiratory failure shortly after birth [105]. Their lungs at birth were severely atelectatic and development was impaired at the early canalicular stage [105]. In FDLE cells of fetal rats, GR mRNA expression was significantly higher in female cells, possibly rendering them more receptive to GCs [106]. In contrast, the total number of lung GR and the measured binding affinity did not significantly differ between male and female sheep fetuses [107].

In conclusion, all steroid receptors are expressed in the fetal lung, although not to the same degree, since ER-β, PR-A and GR-α constitute the dominant isoforms. Importantly, steroid receptor expression differs between multiple lung cell types and developmental stages. It is further regulated by the presence of steroids.

### 2.2. Steroid Metabolism in the Fetal Lung

Sex steroids including androgens (e.g., T, DHT), estrogens (with E2 being the most potent), and P, are all derived from cholesterol. These steroids are synthesized primarily in the gonads, the adrenal gland and the feto-placental unit. Cholesterol is first converted to pregnenolone whereupon the steroidogenic pathway diverges towards the formation of sex steroids, GCs, or mineralocorticoids.

In the sex hormone pathway, pregnenolone is initially converted to P or dehydroepiandrosterone (DHEA) which can be further converted to androgens and estrogens [66]. Testes do not secrete T during the saccular stage when sex differences in fetal lung development were most pronounced. Considerable evidence exists for local production in peripheral tissues including the lung, which depends on the steroidogenic enzymes present within specific tissues [108]. Notably, these nongonadal sources possibly enable local sex steroid production or its metabolism resulting in substantial differences between pulmonary steroid levels compared with levels seen in the circulation [75]. The fetal lung is unlikely to use cholesterol to synthesize androgens and estrogens, because midgestational lungs do not express cytochrome P450 aromatase [109]. Instead, conversion of circulating adrenal precursors, namely DHEA and androstenedione, by steroidogenic enzymes enables pulmonary production of sex steroids [110].

**Figure 3:**
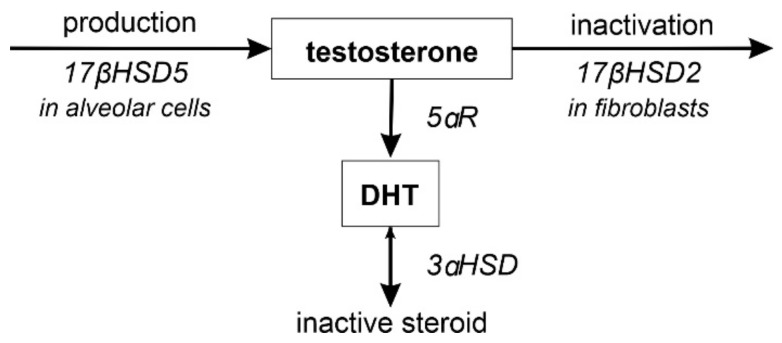
Local lung androgen metabolism. 17βHSD5 results in T production, whereas 17βHSD2 inactivates T. The 5αR reduces e.g., T to DHT, which 3αHSD rapidly inactivates. Production and inactivation of androgens take place in different lung cell types during development.

In the developing lung, an active androgen metabolism with androgen synthesis and inactivation is present [111,112]. Multiple isoforms of the 17β-hydroxysteroid dehydrogenase (17βHSD) exist. Expression of specific 17βHSD isoforms in cells will either result in production of active 17β-hydroxyandrogens (17βHSD types 1, 3, 5 or 7) or inactive 17-ketosteroids (17βHSD2, 4, 6 or 8) [111]. Furthermore, the 3αHSD catalyzes the reversible conversion of 3α-androstanediol to DHT and vice versa, and the 5α-reductase (5αR) reduces e.g., T to DHT. A simplified scheme can be found in Figure 3. In the alveolar cell line A549, 17βHSD5 was shown to convert androstenedione into T [112]. In combination with a low 5αR activity in A549 cells, T accumulation and secretion was achieved [112]. On the other hand, A549 cells not only lack the capacity to generate DHT, but rapidly inactivate it by 3αHSD3 activity [112]. According to the results obtained in A549 cells, a direct effect of DHT seems unlikely and T presumably represents the active androgen in the pulmonary circulation. Expression of the 17βHSD5 and 3αHSD3 has been observed in the developing human and murine lung with temporal changes [101,113]. While very low levels of 3αHSD3 (DHT inactivation) were measured during the canalicular stage, a strong increase was observed in the saccular stage for both sexes [113]. Several lung fibroblast cell lines demonstrated high expression of 17βHSD2 (T inactivation) [111], suggesting that fibroblasts likely inactivate T produced and secreted by alveolar cells [96]. Furthermore, 17βHSD2 expression was also observed in epithelial cells of the developing mouse lung [101]. Notably, fibroblasts also expressed 5αR1, involved in DHT synthesis from T, which however showed no activity on T, thus limiting androgenic effects [111]. Expression of 5αR1 was demonstrated in the canalicular and saccular stage with no difference between sexes [113]. Expression of 17βHSD2 and 5 are both upregulated in correlation with the emergence of mature ATII cells in both murine male and female lungs peaking at the beginning of the saccular stage [96]. No sex differences in 17βHSD5 expression were observed at this stage, but females showed higher 17βHSD5 expression levels during the canalicular stage [96]. No temporal or sex differences for expression of 17βHSD2 have been detected [101]. Besides, 17βHSD2 activities further include inactivation of E2 to estrone, and formation of P [101], although involvement of these enzymatic activities in lung development has not been addressed. Furthermore, 17βHSD1 and 7, which are possibly involved in local E production, are expressed in the fetal lung [114]. Expression of these steroidogenic enzymes strongly suggests a pulmonary steroid metabolism during fetal lung development.

In addition to temporal changes, a further level of complexity is added by local differences in the expression of steroidogenic enzymes. Although studies on this subject are limited, 17βHSD5 protein was observed in some epithelial cells of the conducting zones, while high expression levels of 17βHSD2 were shown in epithelial cells of the budding parts in respiratory ducts [97]. Indeed, synthesis and inactivation must occur in different cells according to local needs and thereby fine-tune androgenic pressure. These observations lead Tremblay and Provost (2013) to propose a model for the control of budding sites by T [115]. According to this hypothetic model, T is produced by 17βHSD5-positive cells of the conducting zones and is secreted in the lumina, resulting in a decreasing gradient from the conducting to the distal zone. The authors further suggest that T may modulate expression of growth regulators in such a way that budding is impaired in the presence of T and becomes possible in the distal areas where T levels become low due to 17βHSD2 activity [115]. Although this model awaits experimental proof, it is nevertheless a highly interesting concept of regional androgen metabolism contributing to the regulation of fetal lung growth.

In addition to a variable expression of steroid receptors, the spatial and temporal differences in steroidogenic enzymes, producing or inactivating steroid hormones, makes it highly complicated to determine estrogenic or androgenic influences during fetal lung development. Moreover, DHT competes with E2 for binding to the cytosolic ER, suggesting that antiestrogenic actions of androgens are not necessarily mediated by the AR [116,117]. Furthermore, a metabolite of DHT stimulated E-dependent cell growth via ERs but inhibited growth via ARs when the ER was occupied by estrogens [118]. Thus, steroid hormones with significant affinity for more than one receptor can exert opposing biological effects in the same cells. Obviously, reporting serum or even pulmonary steroid levels greatly underestimates the complexity of the (sex) steroid metabolism in the fetal lung and impedes conclusions about sex-specific differences therein.

### 2.3. Sex-Specific Differences of Lung Development

#### 2.3.1. Sex-Specific Aspects of Lung Growth

Studies in adult rodents demonstrated that, starting at the age of two months, female rats had higher body mass-specific gas-exchange surface areas and smaller alveoli than age-matched males [119]. Furthermore, early ovariectomized rats (day 21) had smaller body mass-specific gas-exchange surface areas and larger alveoli at the age of two months than sham-ovariectomized rats, which was prevented by E therapy [120]. In agreement, E supplementation in females induced smaller and more numerous alveoli. The authors further reported that treatment of newborn female rats with T did not affect gas-exchange areas or alveolar size. In addition, testicular feminization (AR-deficiency) did not affect gas-exchange areas or alveolar size compared to wild-type littermates [120], although observed animal numbers were limited. The authors concluded that E is the only sex steroid that induces sexual dimorphisms in gas-exchange areas and alveolar size. In newborn piglets, prenatal E deprivation impaired alveolar formation and AFC [121]. In agreement, ER and PR antagonism reduced alveolar counts in pig fetuses and abolished sex differences [122]. Furthermore, female fetal rats contained a higher number of FDLE cells per fetus [72]. The importance of ERs was further addressed in ER-deficient (ERKO) mice. ERKO mice demonstrated that ER-α mediates the sexual dimorphism of gas-exchange areas and alveolar numbers, while both ER-α and ER-β mediate the sexual dimorphism of alveolar size [123]. Thus, ERs regulate alveolar size and number in a nonredundant manner, and E is further required for maintenance of already-formed alveoli and induces alveolar regeneration after alveolar loss in adult ovariectomized mice [124,125]. Furthermore, adult βERKO mice exhibited pulmonary deficiencies in platelet-derived growth factor A (PDGF-A) and granulocyte/macrophage colony-stimulating factor (G/M-CSF) [73], both of which are transcriptionally regulated by ER-β and crucially involved in alveolarization and surfactant production. Notably, defective PDGF receptor signaling represents a feature of BPD development [126]. Moreover, mesenchymal stem cells isolated from tracheal aspirates of premature infants demonstrated that male infants developing BPD expressed significantly lower mRNA and protein levels of PDGF-A receptor, confirming sex differences [127]. In addition, PDGF-A mRNA expression was more responsive to E2 stimulation in female rodent FDLE cells [72]. Vascular endothelial growth factor (VEGF) serum levels are also regulated by E, thus indirectly affecting cellular proliferation [16,128]. In agreement, the lung tissue of fetal pigs demonstrated sex differences in the VEGF mRNA expression, which was abolished by ER & PR antagonism [122]. ER-β is further necessary for maintenance of the extracellular matrix composition and loss of ER-β results in abnormal lung structure and systemic hypoxia [129]. PR activation generally promotes differentiation and inhibits cellular proliferation in contrast to estrogenic effects [130]. However, E demonstrated anti-proliferative effects on lung myofibroblasts involving the MAPK pathway [131]. Organ cultures of fetal rat lungs showed that both DHT and E reduced epithelial cell proliferation only in tissues taken during the rapid growth phase from the late pseudoglandular to early saccular stage [132]. The contrasting results might be due to a biphasic pro-proliferative and anti-proliferative response [133].

Sex differentiation begins at conception with the sex-determining region Y (SRY) gene being transcribed at the 2-cell stage, which triggers growth acceleration in XY embryos. This accelerated growth is supposedly important for the male embryo possibly allowing complete testicular differentiation before levels of estrogens become too high as pregnancy progresses [20]. Early reports demonstrated that androgens induce multiplication of immature ATII cells in vitro without allowing the cells to become mature [134]. Furthermore, DHT treatment resulted in higher fetal lung mass, possibly due to apparent increases in the numbers of lung fibroblasts and ATII cells [134]. In agreement, DHT treatment of fibroblasts and ATII cells in vitro increased cellular proliferation and inhibited maturation of ATII cells [134]. Androgens also increased proliferation of vascular smooth-muscle cells [135]. DHT has been shown to enhance proliferation by up-regulating AR expression involving EGF and p38 MAPK [136]. Notably, DHT binding through AR enhanced structural progression of branching morphogenesis by increasing fibroblast and epithelial proliferation and programmed cell death in the developing mouse [137] and human [100]. Fetal sex did not influence the DHT response as shown in mouse lungs of the early pseudoglandular stage [137]. These studies suggest that male fetal lung differentiation is delayed due to a prolonged androgen-induced growth [134]. Thus, the higher susceptibility of premature male infants to RDS might be the consequence of reduced numbers of mature ATII cells [138,139] and a larger lung size compared with premature female infants [16]. Furthermore, DHT-induced proliferation was not reduced by simultaneous GC treatment [134]. Androgens were reported to exhibit inhibitory effects on antenatal lung development and reduce tissue levels of GR mRNA and protein in fetal rat lungs [19,140], possibly limiting the stimulating effect of antenatal GCs.

During the active growth phase, ERs regulate the alveolar size and number, as well as the maintenance and alveolar regeneration. Androgens, on the other hand, affect proliferation of epithelial, endothelial, and mesenchymal cells in the developing lung.

#### 2.3.2. Sex-Specific Aspects of Lung Maturation

##### Surfactant Synthesis

It is important to note that fetal E levels are similar for male and female fetuses throughout gestation, while T and DHT levels differ [141,142,143]. A microarray analysis of murine fetal lungs demonstrated sex differences or flutamide modulation of AR-interacting genes, genes related to surfactant phospholipid synthesis, and lung developmental regulator genes [144]. Therein, a delay in lung maturation between male and female fetal lungs was noted at the transition between canalicular and saccular stages of lung development, which overlaps the surge of surfactant production, with females pursuing lung maturation while males are not yet fully engaged in differentiation at this period [144]. In agreement, the fetal lung of females has been shown to mature faster as surfactant is produced earlier by females [145,146]. Previous studies in humans showed that pulmonary maturity, assessed by amniotic fluid levels of surfactant phospholipids, was higher in female infants from 30 to 40 weeks of gestation [147]. The difference in the degree of pulmonary maturity was 1.2 to 2.5 weeks with females ahead of males [145,147]. Consistently, the development of surfactant production during the canalicular to early saccular stage was delayed by 1 day in male mice compared to females [148,149]. Sex-specific organ culture of fetal rabbit lungs demonstrated that female lungs synthesized more saturated phosphatidylcholine per mg protein than male lungs and dexamethasone stimulated phospholipid synthesis only in female fetal lungs [150]. No sex differences were observed in the very immature or the mature fetuses [151]. Studies of polytocous mammals reported an association between the sex of neighboring fetuses and fetal surfactant production of females, such that with one or two male neighbors, females showed decreasing surfactant production accompanied by higher androgen plasma levels [152,153]. Both DHT and E reduced surfactant synthesis in early canalicular lung explants, while in midcanalicular explants, DH reduced surfactant synthesis of female explants to male levels [132]. Subsequently, DHT showed no effect on any tissue, but E stimulated surfactant synthesis in both male and female early saccular explants only [132]. Chronic exposure of pregnant dams to DHT further decreased surfactant protein B and C mRNAs in male and female fetal lungs [154]. In pregnant rabbit dams, administration of DHT during the pseudoglandular and canalicular stage reduced fetal surfactant production and eliminated the sex differences by lowering the female to male values. In agreement, administration of the antiandrogen flutamide during the same stages eliminated the sex difference in surfactant levels by increasing the male values to that of females [152]. However, it is important to note that the lowest dose of DHT at which the sex difference has been eliminated was 1 mg per day, resulting in a fourfold increase in plasma androgen levels in females. Although 0.1 mg DHT per day already achieved female plasma androgen levels comparable to male controls, the sex differences persisted [152]. Moreover, in a mouse model of testicular feminization (AR deficiency), the amniotic fluid surfactant phospholipid level of male fetuses was similar to that of females [148]. Sex differences in surfactant lipids were shown to be AR-dependent in fetal male and female mice lungs [155]. Sexed rat littermates exhibited sex differences from ED18-22 with female lungs displaying more lamellar body-containing epithelial cells and more air sacs with lipid material than male littermates [156]. Müllerian inhibiting substance (MIS), a Sertoli cell-derived glycoprotein produced early in testicular ontogenesis, may also inhibit fetal lung development. MIS added to fetal lungs in organ culture suppressed disaturated phophatidylcholine accumulation [157]. These inhibitory effects on biochemical lung maturation observed in vitro were confirmed in vivo [158] and were attributed to a suppression of membrane phosphorylation of EGF receptor (EGFR) by MIS [159]. Notably, testosterone enhances MIS effects [160], possibly contributing to sex-specific effects of this male-specific paracrine factor. In addition, lung β_2_-adrenergic receptor numbers were higher in females compared with males at each gestational age and throughout the neonatal period in rabbits [161]. Notably, the maturation of pulmonary β_2_-adrenergic receptors coincides with the onset of the surfactant flux into the tracheal fluid and both mature more rapidly in female fetal lambs [162]. Based on these results, the maturation of the surfactant system is possibly delayed by androgens in male fetuses.

Lung maturation greatly depends on epithelial–mesenchymal interactions mediated by soluble factors. GCs induce FPF production by lung fibroblasts, which in turn stimulate the alveolar epithelium to synthesize surfactant phospholipids. Notably, only mature fetal rat lung fibroblasts had this FPF-mediated stimulatory effect on fetal ATII cells, whereas immature (pseudoglandular) lung fibroblasts blocked the stimulatory action. This led to the identification of the transforming growth factor (TGF)-β, an endogenous inhibitor of maturation, which is produced by immature mesenchyme inhibiting epithelial maturation [163]. Only after a decrease in TGF-β activity, in later stages of lung development, epithelial “automaturation” proceeds [163]. Notably, chronic androgen treatment of pregnant mice during the pseudoglandular and canalicular stage reduced EGFR activity, whereas TGF-β receptor activity was upregulated in lung fibroblasts of both sexes [154]. Thus, DHT treatment altered the balance of growth factor signaling [154]. GCs have been shown to down-regulate TGF-β and induce FPF production in lung fibroblasts [164]. DHT application to pregnant rats during the pseudoglandular/canalicular stage decreased FPF activity from male and female control and cortisol-treated fetal fibroblasts [165]. It was further shown that DHT blocked the cortisol-induced surfactant synthesis in organotypic cultures, without affecting basal activity [166]. Thereby, DHT inhibited both the cortisol-stimulated FPF production by fetal lung fibroblasts and the FPF activity on surfactant synthesis in ATII cells [166]. When ATII cells were exposed to cortisol-conditioned fibroblast medium with neutralizing TGF-β antibodies, male cells exhibited greater cytidylyltransferase activity, the rate-limiting enzyme for surfactant phosphatidylcholine synthesis, compared with females [167]. This suggests a sex-specific inhibitory role for TGF-β on enzyme activity under GC exposure or that TGF-β may have suppressed GC-induced FPF release more in male fibroblasts [167]. Interestingly, in tracheal aspirates of very preterm infants up to fivefold higher levels of TGF-β were observed [168]. No sex differences in either FPF production or surfactant synthesis by ATII cell were observed during the pseudoglandular stage in rats [165]. Subsequently, a sex difference in FPF production was detected in the late canalicular stage, while ATII cell surfactant synthesis differed at the beginning of the saccular stage, suggesting a delay of approximately 24 h in male fetuses [165]. In agreement, GC-induced production of FPF was shown in fetal female mouse fibroblasts starting at the beginning of the saccular stage, and in males 1 day later [149]. EGF advanced cortisol-induced FPF production such that female fibroblasts produced FPF in the late canalicular stage, while age-matched male fibroblasts remained unresponsive [149]. Subsequently, at the beginning of the saccular stage, fibroblasts from both sexes produced FPF in response to EGF and cortisol [149]. Previous studies suggested that the female fetal lung has more EGF binding sites than the male lung [169,170], possibly explaining the differential responsiveness. Fibroblasts from AR-deficient mice confirmed a sex-specific effect of EGF, which was related to the developmental stage of the fetal lung [149]. Thus, EGF affected the timing of fibroblast maturation and did so in a sex-specific manner.

Conditioned media prepared from fetal rat lung fibroblasts treated with DHT, T, or T plus 4-MA (17β-N,N-diethylcarbamoyl-4-aza-4-methyl-5α-androstane-3-one) exhibited no FPF activity in contrast to untreated fibroblasts [171]. Notably, blocking formation of DHT by application of 4-MA to pregnant rats did not affect the sex differences in surfactant synthesis [171], questioning the physiological relevance of DHT in lung development. However, DHT affected fibroblasts in vitro, possibly attributable to the lack of epithelial cells, which are able to inactivate DHT rapidly through 3αHSD activity [113]. Regarding the expression of steroidogenic enzymes, fibroblasts likely inactivate T produced and secreted by ATII cells, which has been suggested as a possible paracrine factor [96]. The study indicated that the production of androgens by mature ATII cells could down-regulate fibroblast–epithelial cell communication to accelerate cell reprogramming after the emergence of mature ATII cells [96]. In agreement, several studies suggested that androgens exert their negative effect on the surge of surfactant by their action on fibroblasts, because they reduced the fibroblasts’ potential to induce ATII cell maturation through paracrine factors [165,166]. It is important to note that these studies were done using DHT, which is unlikely to be involved in physiological lung development or is rapidly inactivated by ATII cells [112].

**Figure 4:**
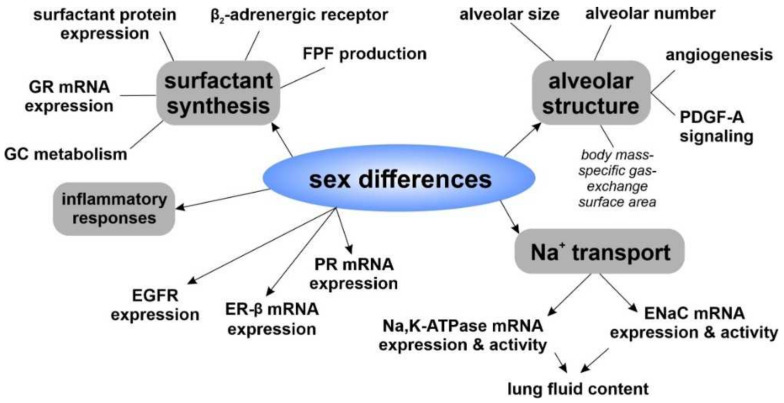
Sex differences in fetal lungs. Studies showed that the fetal sex has multiple effects on the developing alveolar structures, surfactant synthesis and Na^+^ transport, among others.

**Figure 5:**
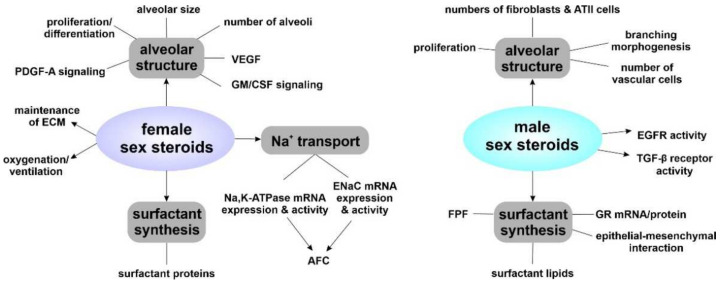
Impact of female and male sex steroids during fetal lung development. A relationship between male and female sex steroids and the developing alveolar structure as well as surfactant synthesis has been reported. In contrast, Na^+^ transport was affected only by female sex steroids.

Another sex difference was noted for the 11-oxidereductase activity, which converts inactive cortisone to active cortisol. Fibroblast cultures of late canalicular stage rats displayed lower 11-oxidereductase activity in males compared to females, indicating lower ability of male fetal fibroblasts to produce active cortisol [172]. Notably, it was shown that sex differences in surfactant phospholipid content were not due to differences in phospholipid turnover, but rather differential regulation of specific metabolic steps within the surfactant synthesis pathway. More precisely, differences in choline transport and the activity of cytidylyltransferase were observed [167]. Figure 4 summarizes findings of sex differences, while Figure 5 outlines the effects of male and female sex steroids during fetal lung development.

##### Alveolar Fluid Clearance

Sexually mature female rats have higher α-ENaC mRNA levels relative to males and administration of E2/P elevates mRNA expression of ENaC α- and γ-subunits in immature female rats [173]. In agreement, female rodent FDLE cells demonstrated higher basal and amiloride-sensitive Na^+^ transport that was underlined by an increased maximal ENaC and Na,K-ATPase activity in females [72]. This was accompanied by higher mRNA levels of the ENaC- and Na,K-ATPase subunits in female-derived FDLE cells [72]. These sex differences suggest a higher or earlier onset of AFC in female rat pups that was corroborated by lower lung wet-to-dry weight ratio in female fetal and newborn rat pups [72]. Importantly, these sex differences in Na^+^ transport were abolished by inhibition of ER-β [72,102]. It has been shown that E2 increases ENaC density and open probability through stimulation of G protein-coupled ER resulting in enhanced ENaC trafficking to the plasma membrane [174]. Furthermore, E induced a rapid-onset and sustained increase of ENaC activity in kidney cortical collecting duct cells, which was blocked by inhibition of PKCδ, metalloproteinase activity, EGFR, phospholipase C and ER activity [175]. Notably, a nuclear-excluded E2 conjugate showed similar stimulatory effects on ENaC activity [175]. An increased number of responsive patches containing putative ENaC activity were observed in FDLE cells induced by E2 and P [176]. Moreover, E2/P dose-dependently elevated basal and amiloride-sensitive Na^+^ transport in FDLE cells [176], especially in female-derived FDLE cells [102]. Baseline AFC was higher in female rats compared to male rats that was attenuated by bilateral ovariectomy, suggesting a female advantage [177]. E2-binding to ER-β further increased N-myc downstream-regulated gene 2 (NDRG2) gene expression, which in turn interacted with and thereby stabilized Na,K-ATPase-β1 by inhibiting its ubiquitination and degradation [178]. This interaction enhanced the Na,K-ATPase-mediated Na^+^ transport in epithelial cells [178]. In contrast, T lacked any effect on Na^+^ transport in male and female FDLE cells [102]. Accordingly, inhibition of the AR by flutamide did not abolish the sex difference in Na^+^ transport, further supporting the view that fetal alveolar epithelial Na^+^ transport is largely unaffected by androgens [102]. In contrast, AR and ENaC are regulated by androgens in the kidney, with DHT decreasing expression of all ENaC subunits in female rats, while T showed only a trend towards a lower ENaC expression [92]. As discussed above, sex differences in EGF and GC signaling have been noted. Analyzing their effect on Na^+^ transport demonstrated that chronic EGF treatment reduced ENaC mRNA levels and activity in both male and female FDLE cells [179]. In contrast, acute effects of EGF were sex-specific with a reduced Na^+^ transport observed only in male FDLE cells [179]. This sex-specific differential response to acute EGF application was suggested to be due to increased AKT phosphorylation in females, while pERK1/2 was elevated in both male and female FDLE cells [179]. Nevertheless, the study suggested that EGF unlikely represents the cause for the sex differences in Na^+^ transport. Furthermore, GCs increased ENaC activity and mRNA expression in FDLE cells, independent of sex and inhibition of GR did not equalize Na^+^ transport between male and female cells, suggesting that GR activity does not contribute to the increased Na^+^ transport in females [106].

## 3. Conclusions

Besides differences in alveolar structure between male and female fetal lungs, fetal lung maturation also differs in several aspects. Differences in the onset of surfactant synthesis were corroborated by alterations of the epithelial-mesenchymal relationship, possibly mediated by sex steroids. Moreover, epithelial Na^+^ transport, crucially involved in perinatal lung transition, was shown to be sex-specific. According to the presented evidence, a prolonged growth phase for male fetuses and thus a later onset of maturation possibly results in the “male disadvantage”. However, the major issue in studying sex differences in lung growth and maturation is the crosstalk and interaction between steroids. Local steroid metabolism and conversion such as aromatization of T resulting in E2 and differential steroid receptor expression with opposing actions, such as ER antagonizing ER function or PR-B antagonizing PR- and ER, add further complexity. Moreover, steroids differentially modulate their own receptor expression and that of other steroid receptors. Thus, understanding the crosstalk and interaction is essential for understanding sex differences in pulmonary complications.
